# Glucocorticoids promote neural progenitor cell proliferation derived from human induced pluripotent stem cells

**DOI:** 10.1186/2193-1801-3-527

**Published:** 2014-09-15

**Authors:** Eiichi Ninomiya, Taeka Hattori, Masashi Toyoda, Akihiro Umezawa, Takashi Hamazaki, Haruo Shintaku

**Affiliations:** Department of Pediatrics, Osaka City University Graduate School of Medicine, 1-4-3 Asahi-machi, Abeno-ku, Osaka, 545-8585 Japan; Research Team for Geriatric Medicine (Vascular Medicine), Tokyo Metropolitan Institute of Gerontology, Sakaecho 35-2, Itabashi-Ku, Tokyo, 173-0015 Japan; Department of Reproductive Biology, National Research Institute for Child Health and Development, 2-10-1 Ookura, Setagaya-ku, Tokyo, 157-8535 Japan

**Keywords:** Glucocorticoids, Neural progenitor cell, iPSC, Cell culture, Proliferation

## Abstract

Glucocorticoids (GCs) are frequently used for treating and preventing chronic lung disease and circulatory dysfunction in premature infants. However, there is growing concern about the detrimental effects of systemic GC administration on neurodevelopment. The first choice of GCs to minimize the adverse effects on the developing brain is still under debate. We investigated the effect of commonly used GCs such as dexamethasone (DEX), betamethasone (BET) and hydrocortisone (HDC) on the proliferation of human-induced pluripotent stem cell (iPSC)-derived neuronal progenitor cells (NPCs). In this study, NPCs were treated with various concentrations of GCs and subjected to cell proliferation assays. Furthermore, we quantified the number of microtubule-associated protein 2 (MAP2) positive neurons in NPCs by immunostaining. All GCs promoted NPC proliferation in a dose-dependent manner. We also confirmed that MAP2-positive neurons in NPCs increased upon GC treatment. However, differential effects of GCs on MAP2 positive neurons were observed when we treated NPCs with H_2_O_2_. The total numbers of NPCs increased upon any GC treatment even under oxidative conditions but the numbers of MAP2 positive neurons increased only by HDC treatment. GCs promoted human iPSCsâ€“derived NPC proliferation and the differential effects of GCs became apparent under oxidative stress. Our results may support HDC as the preferred choice over DEX and BET to prevent adverse effects on the developing human brain.

**Electronic supplementary material:**

The online version of this article (doi:10.1186/2193-1801-3-527) contains supplementary material, which is available to authorized users.

## Introduction

In recent years, systemic glucocorticoids (GCs) have frequently been administered to treat and prevent chronic lung disease (CLD), which is also known as bronchopulmonary dysplasia, and circulatory dysfunction in premature infants. GCs administration dramatically improves the outcome of premature infants with established CLD (Halliday et al. 2009, 2010). However, the use of GCs, especially dexamethasone (DEX), for CLD patients is reported to show detrimental effects on the developing brain with subsequent behavioral alterations and cerebral palsy (Murphy et al. 2001; Shinwell et al. 2000; Yeh et al. 2004). Betamethasone (BET) is also reported to impair cerebral blood flow velocities in very premature infants with severe CLD (Cambonie et al. 2008). A few studies have suggested that patients treated with HDC showed no neurological adverse effect (Benders et al. 2009; de Jong et al. 2011; Watterberg et al. 2007), but another study showed that GCs reduced proliferation and induce differentiation of neurons (Aden et al. 2011). The effect of GCs on the developing human brain remains elusive, and randomized clinical trials are required in order to establish better neurological outcomes.

Many studies have been conducted to reveal the mechanisms underlying the adverse effects of GCs. DEX treatment has been shown to decrease brain weight (Kanagawa et al. 2006) and inhibit hippocampal neurogenesis (Kim et al. 2004) in rats. In addition, DEX inhibited the proliferation of embryonic rat neural stem cells (Bose et al. 2010). Duksal et al. (2009) reported that high dose DEX caused brain weight loss due to neuronal apoptosis. Although many animal studies have indicated that GCs suppress the proliferation of neuronal cells, it remains unknown how GCs affect neuronal cells in humans.

In the present study, we investigated the effects of commonly used GCs such as DEX, BET and HDC on the proliferation of human iPS cell-derived NPCs, which were used as a model of human embryonic and neonatal NPCs. We further focused on the subpopulation of NPCs that were committed to the neuronal lineage. The effects of GCs on neural cell proliferation were evaluated. We also examined whether oxidative stress affected the sensitivity of NPCs to GCs.

## Methods

### Reagents

DECADRON® (Dexamethasone) was obtained from MSD (Tokyo, Japan). Rinderon® (Betamethasone) was from Shionogi & Co., Ltd. (Osaka, Japan). Hydrocortone® (hydrocortisone) was from Nichi-Iko Pharmaceutical Co., Ltd. (Toyama, Japan). Hydrogen peroxide solution (H_2_O_2_) was from Wako Pure Chemical Industries, Ltd. (Osaka, Japan).

### Human iPS cell culture and neural progenitor cells induction

The study was approved by the Ethics Committees of the Osaka City University (approval #2472) and was conducted according to the declaration of Helsinki. In this study, we used human iPSCs derived from fetal lung fibroblast (MRC-5) cells and the iPSCs were maintained by standard culture methods as described previously (Saito et al. 2011). Neural induction was performed as described previously (Chambers et al. 2009). Briefly, neural induction will be initiated by 10 μM SB431542 (TGF-β inhibitor, Wako) and 200 ng/ml of Noggin (R&D Systems, Minneapolis, MN). After 8 days of neural induction, cells are dissociated with accutase (Chemicon, Temecula, CA) and plated onto poly-ornithine and laminin (Sigma, St. Louis, MO) with neurobasal medium supplemented with 2% B27 (Invitrogen), 20 ng/ml bFGF (Wako), 20 ng/ml epidermal growth factor (EGF, Invitrogen). Rosette neural stem cells (R-NSC) will form within a few days. R-NSCs were enriched by Neural Rosette Selection Reagent® (Stem Cell Technologies, Toronto, Canada). NPCs were obtained after a few passages and subjected to proliferation assays. Schematic diagram of induction of NPCs and representative growth rate of NPCs are shown in Additional file 1: Figure S1.

### Proliferation assay

Cell proliferation was measured using Cell Titer 96 AQueous One Solution cell proliferation assay according to the manufacturer’s protocol (Promega, Madison, WI). Ninety-six-well tissue culture plates were coated with poly-ornithine and laminin. NPCs were plated at a density of 6 × 10^3^ cells per well. GCs treatment was started 48 h after plating. After 4 days of GCs exposure, proliferation assays was performed by adding Cell Titer 96 AQueous One Solution and incubating at 37°C for 2 h. Then absorbance was measured at 450 nm with a micro plate reader (MTP-300:CORONA ELECTRIC).

### Immunocytochemistry and MAP2 positive cell count

Chamber slides, µ-slide IV (Ibidi, Martinsried, Germany) were coated with poly-ornithine and laminin. NPCs were plated in the chamber slides at a density of 1.8 × 10^4^ cells per well and cultured for 48 h, followed by exposure to GCs. After 4 days incubation, cells were subjected for immunostaining. Mouse monoclonal anti-MAP2 antibody (AP20) (Chemicon, Temecula, CA) (1:200), Alexa Fluor® 488 Goat Anti-Mouse IgG (Invitrogen) (1:1000), and 4,6-diamidino-2-phenylindole (DAPI) (Sigma) were used. For quantification of MAP2 positive cells and DAPI positive cells, 5 microscopic fields were randomly selected and cells were automatically counted using ImageJ (Schneider et al. 2012). Cell counts per field were standardized against untreated cell counts for each experiment.

### Statistical analysis

For statistical analysis, data were evaluated by analysis of variance (Statcel3, add-in software to Microsoft® Excel 2007). Differences between groups were analyzed by single-factor ANOVA with Tukey-Kramer. Results are displayed with mean ± SD. *P* values < 0.05 were considered statistically significant. All experiments were repeated more than three times.

## Results

### GC treatment promoted neural progenitor cell proliferation

**Figure 1:**
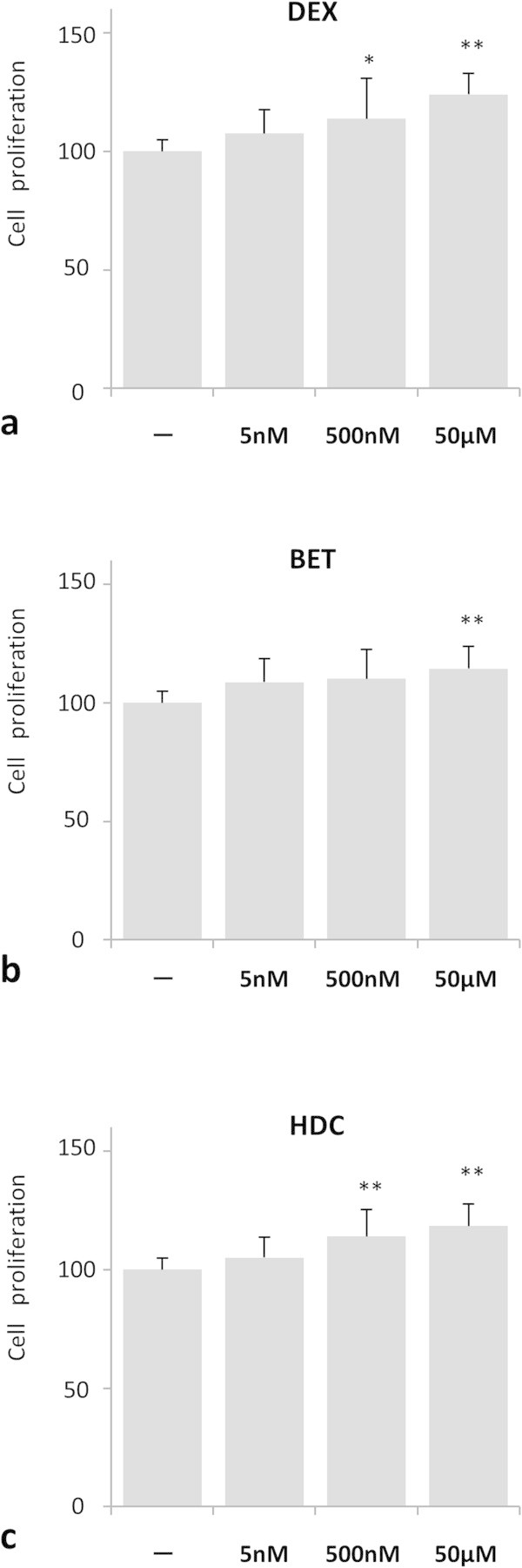
**Glucocorticoid (GC) treatment promoted NPC proliferation.** Cell proliferation was measured by absorbance using Cell 96 AQueous One Assay kit. The average absorbance data were expressed as percentages of untreated samples. *P* values were calculated by comparing with untreated samples (n = 3). *P < 0.05, **P < 0.01. (One-way ANOVA with Tukey-Kramer). The cells were treated with the indicated concentration of **(a)** dexamethasone (DEX), **(b)** betamethasone (BET), and **(c)** hydrocortisone (HDC).

To evaluate the effect of GCs on the proliferation of NPCs, we initially performed a cell proliferation assay. NPCs were exposed to GCs for 4 days and subjected to a proliferation assay. As shown in Figure 1a, the average absorbance of the samples treated with DEX of 5 nM, 500 nM, and 50 µM were 107.5 ± 10.2 (*P* value = NS), 113.8 ± 17.1 (*P* value < 0.05), and 124.0 ± 8.9 (*P* value < 0.01), respectively. The samples treated with BET of 5 nM, 500 nM, and 50 μM were 108.7 ± 9.8 (*P* value = NS), 110.2 ± 12.4 (*P* value = NS), and 114.4 ± 9.4 (*P* value < 0.01), respectively (Figure 1b). The samples treated with HDC of 5 nM, 500 nM, and 50 μM were 105.0 ± 8.6 (*P* value = NS), 114.0 ± 11.3 (*P* value < 0.01), and 118.4 ± 9.3 (*P* value < 0.01), respectively (Figure 1c). We also calculated the *P* values for comparison of each GC from 5 nM to 50 μM. Both DEX and HDC showed statistically significant differences on the absorbance between 5 nM and 50 μM (*P* value < 0.01).

In summary, our results indicate that the GCs stimulate proliferation of NPCs in a dose-dependent manner. Moreover, the proliferative effect was independent from the types of GCs we tested.

### GC treatment promoted cell proliferation of MAP2 positive neuron

GC treatment is reported to increase apoptosis in distinct neural regions in the brain. The studies indicate that neuronal cells are more susceptible than glial cells (Duksal et al. 2009; Hassan et al. 1996). Since iPS cell-derived NPCs consist of heterogeneous populations, we focused on proliferation of the cells that were committed to the neuronal lineage in the GC-treated NPCs.

**Figure 2:**
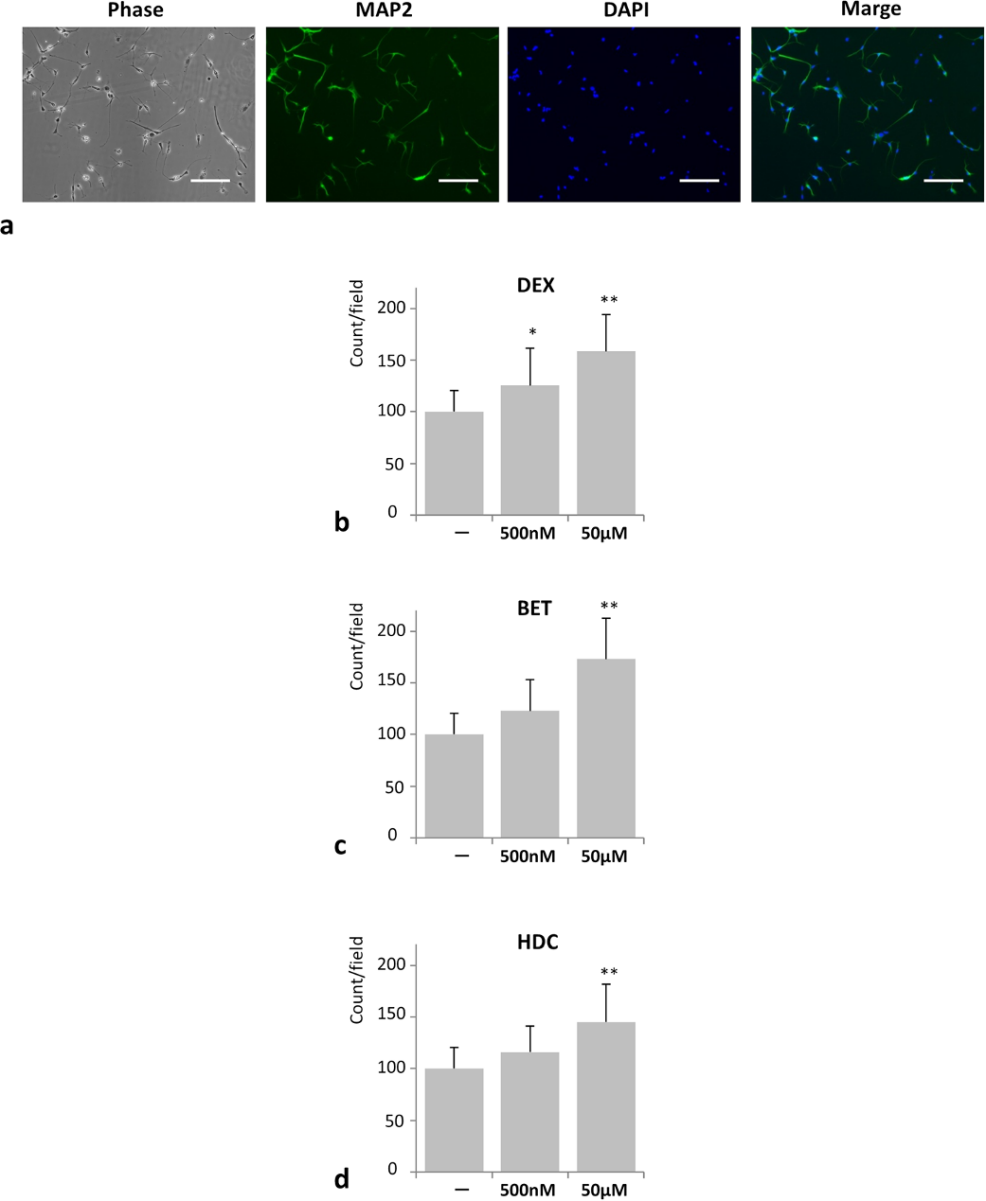
**GC treatment promoted cell proliferation of MAP2 positive neurons.** **(a)** Representative pictures of NPCs stained with an antibody against MAP2 (red) and nuclear counterstain DAPI (blue). Phase, phase contrast image. Scale bar, 100 μm. **(b-d)**. Quantification of MAP2 positive neurons using ImageJ. *P* values were calculated by comparing GC treated with untreated samples (n = 3). *P < 0.05, **P < 0.01. (One-way ANOVA with Tukey-Kramer). The cells were treated with the indicated concentration of **(b)** dexamethasone (DEX), **(c)** betamethasone (BET), and **(d)** hydrocortisone (HDC).

Microtubule-associated protein 2 (MAP2) is specifically expressed *in vivo* in the granular layer in the embryo (Tucker et al. 1989). We performed immunostaining using an anti-MAP2 antibody to evaluate the number of neuronal lineage cells after GC treatments (Figure 2c). For this experiment, NPCs were exposed to GCs for 4 days and then subjected to analysis.

We compared the MAP2 positive cell count with the untreated NPCs and calculated the *P* values. As shown in Figure 2b, the average numbers of MAP2-positive neurons treated with DEX of 500 nM and 50 μM were 125.4 ± 36.0 (*P* value < 0.05) and 158.6 ± 35.3 (*P* value < 0.01), respectively. The MAP2-positive neurons treated with BET of 500 nM and 50 μM were 122.7 ± 36.0 (*P* value = NS) and 173.0 ± 39.6 (*P* value < 0.01), respectively (Figure 2c). The MAP2-positive neurons treated with HDC of 500 nM and 50 μM were 116.0 ± 26.1 (*P* value = NS) and 145.1 ± 36.7 (*P* value < 0.01), respectively (Figure 2d). All GCs showed statistically significant differences on the average numbers of MAP2-positive neurons between 5 nM and 50 μM (DEX and HDC showed *P* value < 0.05, BET showed *P* value < 0.01). These data indicate that the MAP2 positive cell number significantly increased as the cells were treated with a higher dose of GCs. Moreover, we found no significant differences in proliferative potency between DEX, BET, and HDC.

### GC treatment promoted NPC proliferation under oxidative stress

Involvement of oxidative stress was suggested in the pathogenesis of neonatal CLD (Ogihara et al. 1999) and oxidative stress is thought to be a cause of neuronal damage (Ikonomidou and Kaindl 2011). To mimic clinical situations during the use of GCs, we treated NPCs with H_2_O_2_ for oxidative stress and examined the effect of GCs on NPC proliferation. We have initially tested various concentration of H2O2 on the NPCs without GCs. 300 μM H2O2 concentration was reasonably seen the effect of the stress on cellular proliferation (Additional file 2: Figure S2).

**Figure 3:**
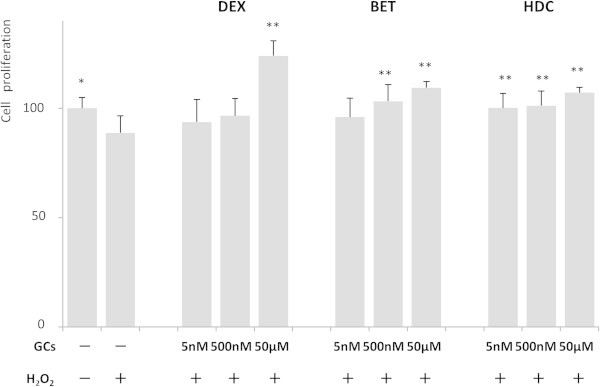
**GC treatment promoted NPC proliferation under oxidative stress.** Cell proliferation was measured by absorbance using Cell 96 AQueous One Assay kit. The average absorbance data were expressed as percentages of untreated samples. *P* values were calculated by comparing GC treated with untreated samples (n = 3). *P < 0.05, **P < 0.01. (One-way ANOVA with Tukey-Kramer). DEX: dexamethasone, BET: betamethasone, HDC: hydrocortisone.

NPCs were treated with 300 μM H_2_O_2_ 1 day before GC treatment. Similarly, we expressed the average  absorbance data as percentages of untreated samples, and the mean percent ± SD of untreated samples was 100 ± 5.0. The results are shown in Figure 3. The average absorbance of cells that were treated only with H_2_O_2_ was 88.8 ± 7.8, which is significantly reduced relative to the untreated samples (*P* value < 0.05, Figure 3).

Initially, we compared the average absorbance with only H_2_O_2_ treated NPCs and calculated the *P* values (Figure 3). The average absorbance of the samples treated with DEX of 5 nM, 500 nM, and 50 μM under H_2_O_2_-treated condition were 93.8 ± 10.3 (*P* value = NS), 96.6 ± 8.0 (*P* value = NS), and 124.0 ± 6.9 (*P* value < 0.01), respectively. The average absorbance of cells treated with BET of 5 nM, 500 nM, and 50 μM under H_2_O_2_-treated condition were 96.0 ± 8.6 (*P* value = NS), 103.2 ± 7.6 (*P* value < 0.01), and 109.4 ± 2.9 (*P* value < 0.01), respectively. The average absorbance of cells treated with HDC of 5 nM, 500 nM, and 50 μM under H_2_O_2_-treated condition were 100.2 ± 6.6 (*P* value < 0.01), 101.2 ± 6.8 (*P* value < 0.01), and 107.2 ± 2.5 (*P* value < 0.01), respectively. When we compared the average absorbance between each GC in the same concentration with or without H_2_O_2_, we did not observe statistical significance except for DEX at 5 nM and 500 nM (*P* value < 0.01). These data indicate that the absorbance significantly increased as the cells were treated with a higher dose of GCs even under H_2_O_2_-treated condition.

In summary, our results indicate that GCs stimulated the proliferation of NPCs under H_2_O_2_-treated conditions. All examined GCs induced NPC proliferation in a dose dependent manner regardless of oxidative stress.

### HDC alone promoted cell proliferation of MAP2 positive neuron under oxidative stress

**Figure 4:**
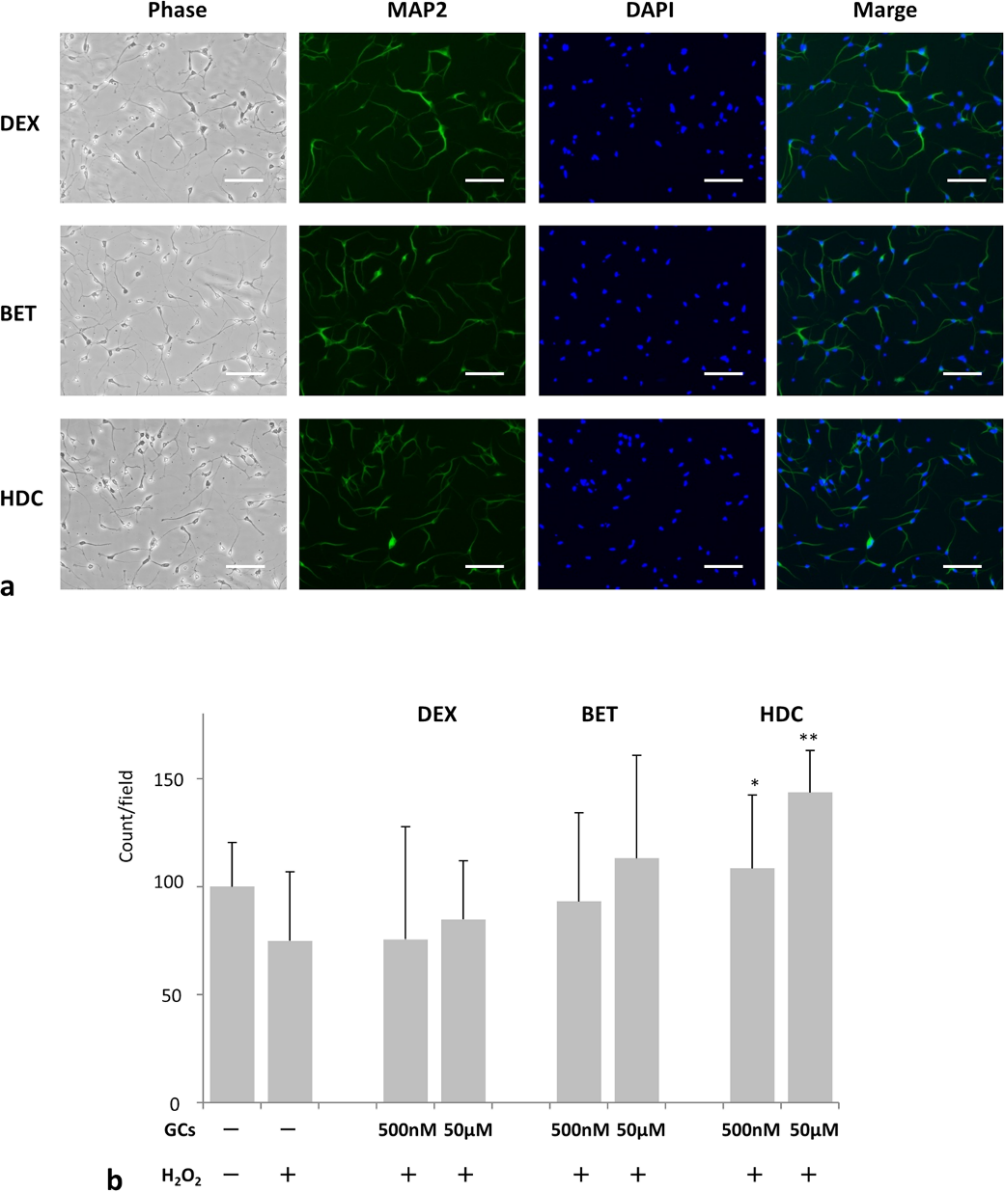
**GCs showed different effects on proliferation of the MAP2-positive neurons under H**_**2**_ **O**_**2**_ **-treated conditions.** **(a)** Representative pictures of NPCs were stained with an antibody against MAP2 (red) and nuclear counterstain DAPI (blue). The cells were pre-exposed to H_2_O_2_ and treated with 50 μM of the indicated GCs. Phase, phase contrast image. Scale bar, 100 μm. **(b)** Quantification of MAP2 positive neurons using ImageJ. *P* values were calculated by comparing GC treated samples with the samples treated with H_2_O_2_ alone (n = 3). *P < 0.05, **P < 0.01. (One-way ANOVA with Tukey-Kramer). DEX: dexamethasone, BET: betamethasone, HDC: hydrocortisone. Note that HDC alone promoted significant cell proliferation of MAP2 positive neurons under oxidative stress.

Neurons are more sensitive to oxidative stress than any other type of cells in the brain (Hayashi et al. 2012). Therefore, we examined the effects of the GCs on the proliferation of MAP2-positive neurons under an oxidative stress condition. NPCs were treated with 300 μM H_2_O_2_ 1 day before GC treatment, then NPCs were exposed to GCs for 4 days and subjected to immunostaining using an anti-MAP2 antibody (Figure 4a).

We compared the MAP2 positive cell count with the condition treated with H_2_O_2_ alone (74.9 ± 31.8). The average numbers of MAP2-positive neurons treated with DEX of 500 nM and 50 μM were 75.6 ± 52.3 (*P* value = NS) and 84.7 ± 27.2 (*P* value = NS), respectively. The average numbers of MAP2-positive neurons treated with BET of 500 nM and 50 μM were 93.1 ± 41.1 (*P* value = NS) and 113.1 ± 47.7 (*P* value = NS), respectively. The average numbers of MAP2-positive neurons treated with HDC of 500 nM and 50 μM were 108.5 ± 34.0 (*P* value < 0.05) and 143.6 ± 19.4 (*P* value < 0.01), respectively. Unlike DEX and BET, HDC significantly increased the number of MAP2-positive neurons compared with the untreated samples even under the H_2_O_2_-treated condition.

In conclusion, only HDC promoted significant cell proliferation of MAP2 positive neurons as well as the total number of NPCs under oxidative stress. DEX and BET, however, increased the total number of NPCs without increasing MAP2 positive neurons.

## Discussion

In this study, we investigated the effect of GCs on proliferation of NPCs derived from human iPS cells. Unexpectedly, all GCs we tested induced NPC proliferation in a dose dependent manner. We also confirmed that MAP2 positive neuronal cells were increased by GC treatment. Furthermore, we investigated the proliferative effects of GCs under an oxidative stress condition that could be more relevant to the clinical setting. The findings revealed that all GCs stimulated the total number of NPCs even under the oxidative condition, but MAP2 positive neurons were only increased by HDC treatment. Our results support the finding that HDC would be the preferred choice over DEX and BET to prevent adverse effects on the developing brain.

### Cell cycle regulation induced by GCs

Samarasinghe et al. (Samarasinghe et al., 2011) demonstrated that the binding of GCs to the GC receptor (GR) decreased gap junction-mediated intercellular communication and led to a decrease in the rate of cells in the S phase. They concluded that GC suppresses cell proliferation through this mechanism. Sundberg et al. (2006) reported that the activation of GR prevents cyclin D1-mediated cell cycle progression and that the high dose GC inhibits proliferation of rat embryonic NPCs. Similar inhibitory mechanisms of GCs were reported by others using rodent cells (Bose et al. 2010). Moors et al. (2012) examined the effect of DEX on neurospheres derived from a 16-week human aborted fetus and found that DEX inhibits the proliferation of human NPCs. However, in our experiment using NPCs derived from human iPS cells, all GCs we tested including DEX intriguingly promoted the proliferation of NPCs.

### Neuroprotective effects of GCs

GCs exhibit protective effects on postmitotic neurons. Harms et al. (2007) showed that GCs induce phosphatidylinositol 3-Akt-kinase-dependent phosphorylation of p21Waf1/Cip1 and it works as a novel anti-apoptotic pathway for postmitotic primary cortical  neurons isolated from rats and mice. By *in vivo* and *in vitro* studies using the rat model, Jeanneteau et al. (2008) showed that GCs activate the Trk neurotrophin receptor and thus exhibit neuroprotective effects. In any case, neuroprotective pathways in postmitotic neurons prevent new cell cycles. Therefore, such a neuroprotective mechanism alone cannot explain how GCs induce NPC proliferation.

### Differential mechanisms of action between GCs

We demonstrated that GCs differently stimulate the proliferation of MAP2-positive neurons under oxidative stress. HDC physically associates with both GR and mineralcorticoid receptor (MR) *in vivo,* while DEX and BET physically associate only with GR but not with MR (De Kloet et al. 1998).  As described above, it has been reported that GR suppresses cell proliferation and causes apoptosis. In the present study, the GCs showed similar effects under non-stressed conditions. Under oxidative stress conditions, however, HDC alone increased the number of MAP2 positive neurons. This may suggest that the activation of MR plays an important role in the proliferation of neurons under oxidative stress. Another possibility could be the difference in the inactivation mechanisms of the GCs. While HDC is metabolized by 11βHSD2, DEX and BET are not sensitive to inactivation by 11βHSD2 (Heine and Rowitch 2009; Noguchi et al. 2011). Thus, the continuous GR activity may induce apoptosis under stress conditions. The human NPCs used in this study were indeed expressing both MR and GR (Additional file 3: Figure S3).

### Clinical implications

Our current study demonstrates that NPCs proliferate in response to GCs but MAP2 positive neurons are sensitive to oxidative stress. It is interesting that the response to HDC is less affected by oxidative stress than DEX or BET (Figure 4). Halliday et al. (2009, 2010) recommended avoiding frequent use of GCs for CLD treatment but there is insufficient evidence regarding which types of GCs to use in order to minimize adverse neurological outcomes. Further clinical and mechanistic studies are required to determine the optimal choice of GCs for children with CLD.

In conclusion, we evaluated the effect of GCs on NPC derived human iPS cells and found unique proliferative effects on NPCs, which were altered by external stress. Further mechanistic studies are needed to reveal how GCs induce NPC proliferation and how oxidative stress modulates the effects of GCs.

## Electronic supplementary material

Additional file 1: Figure S1: Derivation of neural progenitor cells (NPCs) from human iPS cells. (a) Schematic diagram of induction of NPCs. R-NSC: Rosette neural stem cells. (b) Representative picture of R-NSC and NPCs in phase contrast image. R-NSC stained positive for Nestin (green/insert). Bar:100 μm. (c) Growth curve for NPCs. Cell number was automatically measured by using IncuCyte imaging system (Essen BioScience, K.K., Japan). (TIFF 2 MB)

Additional file 2: Figure S2: Effect of H_2_O_2_ treatment on NPC proliferation under oxidative stress. NPCs were treated various concentration of H_2_O_2_ as indicated. Cell proliferation was measured by absorbance using Cell 96 AQueous One Assay kit. The average absorbance data were expressed as percentages of untreated samples. (TIFF 94 KB)

Additional file 3: Figure S3: Expression of glucocorticoid receptor and mineral corticoid receptor in NPCs Quantitative RT-PCR analysis was performed on MRC5-iPSC and NPCs. The mRNA values were expressed relative to the control gene (β-actin). GR: glucocorticoid receptor, MR: mineral corticoid receptor. (TIFF 102 KB)
