# Supplementary figures and images for: Glucocorticoids promote neural progenitor cell proliferation derived from human induced pluripotent stem cells
Source: Springerplus. 2014 Sep 15;3:527. doi: 10.1186/2193-1801-3-527 (PMC4174547; doi:10.1186/2193-1801-3-527)

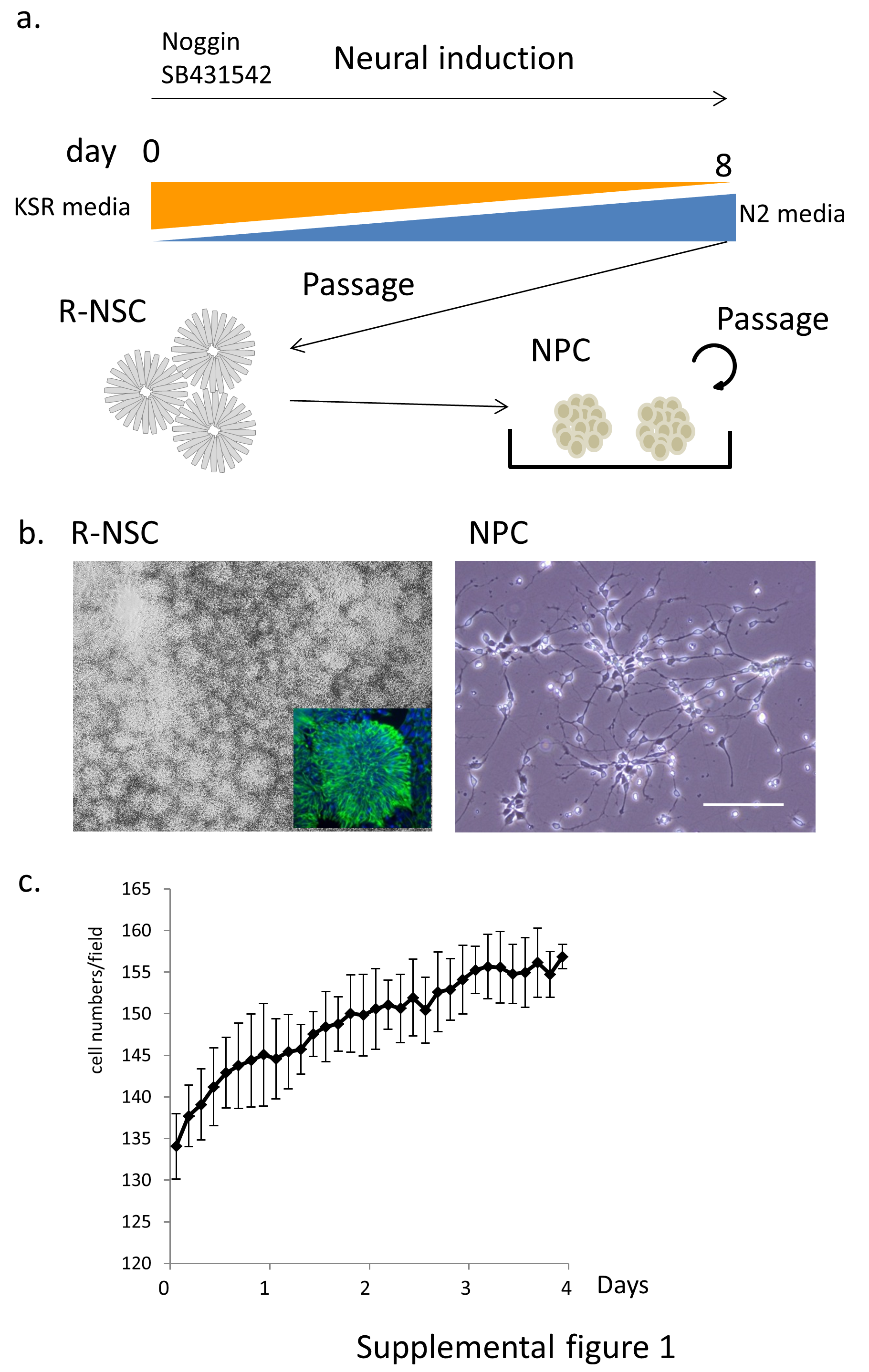

Supplement: Supplementary file 1 — Additional file 1: Figure S1: Derivation of neural progenitor cells (NPCs) from human iPS cells. (a) Schematic diagram of induction of NPCs. R-NSC: Rosette neural stem cells. (b) Representative picture of R-NSC and NPCs in phase contrast image. R-NSC stained positive for Nestin (green/insert). Bar:100 μm. (c) Growth curve for NPCs. Cell number was automatically measured by using IncuCyte imaging system (Essen BioScience, K.K., Japan). (TIFF 2 MB) [file 40064_2014_1233_MOESM1_ESM.tiff]

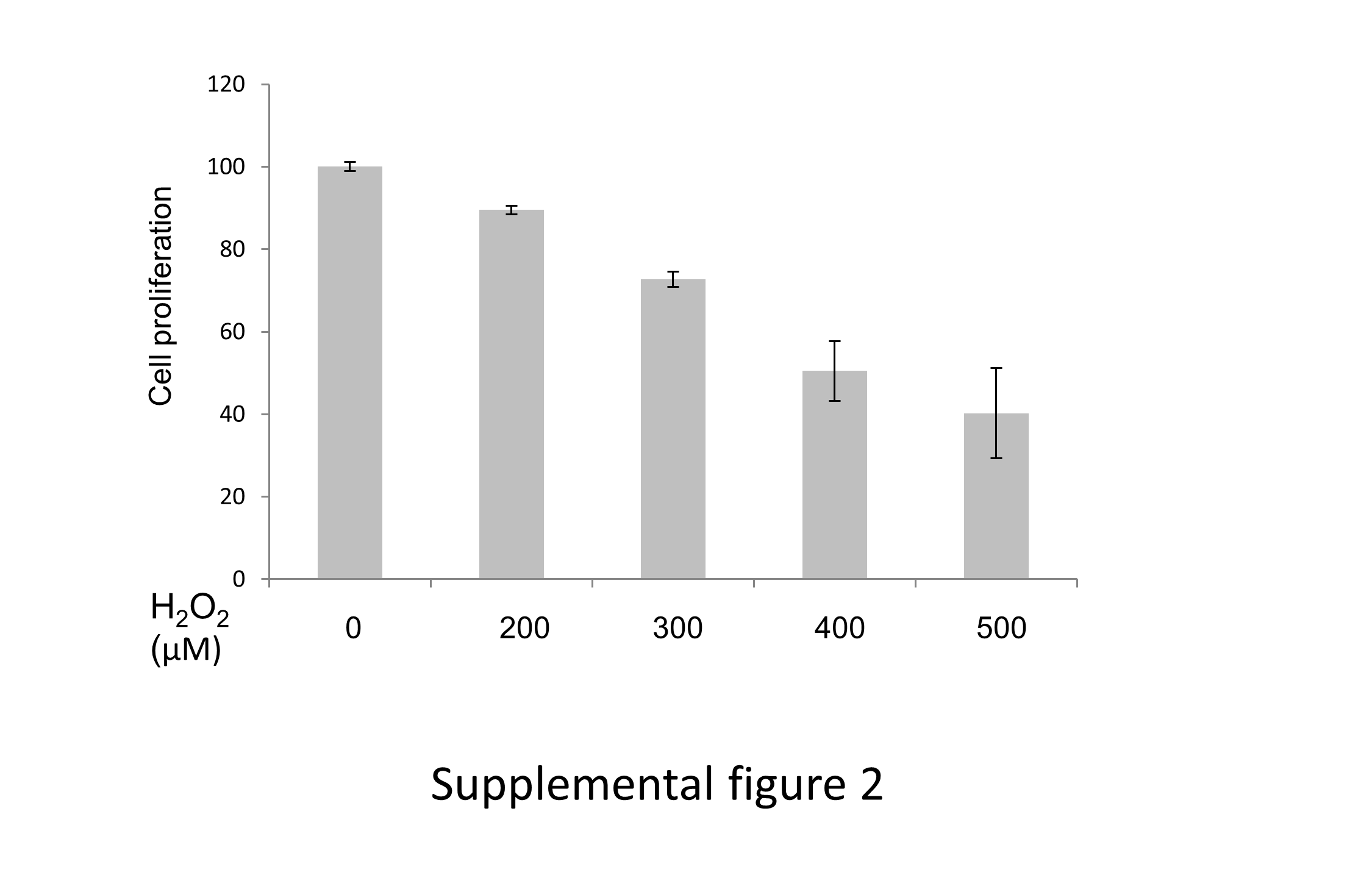

Supplement: Supplementary file 2 — Additional file 2: Figure S2: Effect of H2O2 treatment on NPC proliferation under oxidative stress. NPCs were treated various concentration of H2O2 as indicated. Cell proliferation was measured by absorbance using Cell 96 AQueous One Assay kit. The average absorbance data were expressed as percentages of untreated samples. (TIFF 94 KB) [file 40064_2014_1233_MOESM2_ESM.tiff]

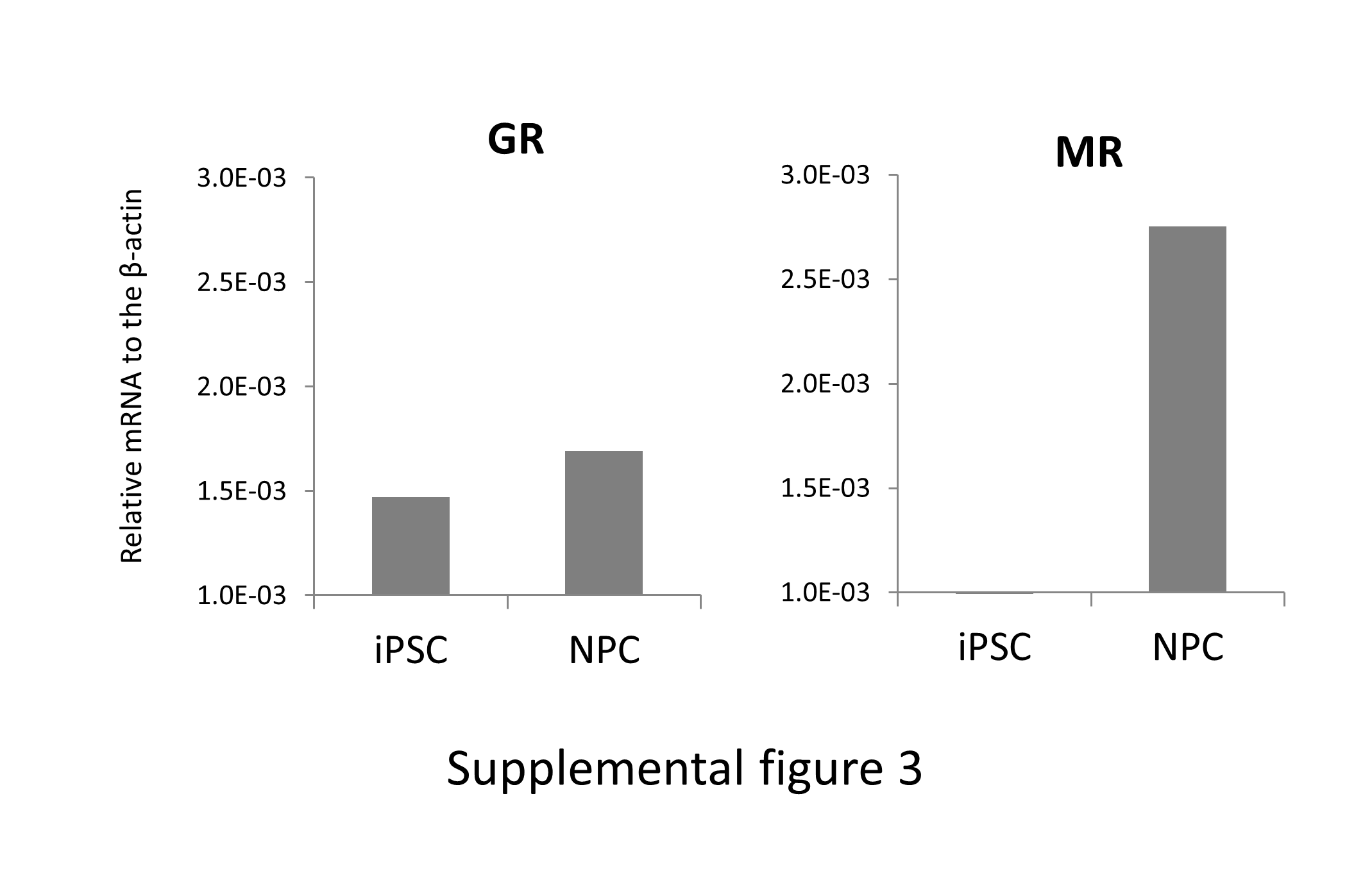

Supplement: Supplementary file 3 — Additional file 3: Figure S3: Expression of glucocorticoid receptor and mineral corticoid receptor in NPCs Quantitative RT-PCR analysis was performed on MRC5-iPSC and NPCs. The mRNA values were expressed relative to the control gene (β-actin). GR: glucocorticoid receptor, MR: mineral corticoid receptor. (TIFF 102 KB) [file 40064_2014_1233_MOESM3_ESM.tiff]
